# Circadian phase-shifting effects of a laboratory environment: a clinical trial with bright and dim light

**DOI:** 10.1186/1740-3391-3-11

**Published:** 2005-09-09

**Authors:** Shawn D Youngstedt, Daniel F Kripke, Jeffrey A Elliott, Katharine M Rex

**Affiliations:** 1 Department of Exercise Science, Norman J. Arnold School of Public Health, University of South Carolina, Columbia, SC 29208, USA; 2 Department of Psychiatry and Sam and Rose Stein Institute for Research on Aging, University of California, San Diego, USA

## Abstract

**Background:**

Our aims were to examine the influence of different bright light schedules on mood, sleep, and circadian organization in older adults (n = 60, ages 60–79 years) with insomnia and/or depression, contrasting with responses of young, healthy controls (n = 30, ages 20–40 years).

**Methods:**

Volunteers were assessed for one week in their home environments. Urine was collected over two 24-hour periods to establish baseline acrophase of 6-sulphatoxymelatonin (aMT6s) excretion. Immediately following home recording, volunteers spent five nights and four days in the laboratory. Sleep periods were fixed at eight hours in darkness, consistent with the volunteers' usual sleep periods. Volunteers were randomly assigned to one of three light treatments (four hours per day) within the wake period: **(A) **two hours of 3,000 lux at 1–3 hours and 13–15 hours after arising; **(B) **four hours of 3,000 lux at 6–10 hours after arising; **(C) **four hours of dim placebo light at 6–10 hours after arising. Lighting was 50 lux during the remainder of wakefulness. The resulting aMT6s acrophase was determined during the final 30 hours in the laboratory.

**Results:**

Neither mood nor total melatonin excretion differed significantly by treatment. For the three light treatments, significant and similar phase-response plots were found, indicating that the shift in aMT6s acrophase was dependent upon the circadian time of treatment. The changes in circadian timing were not significantly correlated to changes in sleep or mood.

**Conclusion:**

The trial failed to demonstrate photoperiodic effects. The results suggest that even low levels of illumination and/or fixed timing of behavior had significant phase-shifting effects.

## Introduction

Older adults have an altered synchronization of circadian rhythms compared to young adults [1-4]. Circadian misalignment of rhythms or malsynchronization might contribute to many age-related disorders of sleep or mood, as has been observed in conjunction with shift-work and jet lag.

It has been hypothesized that age-related circadian malsynchronization might be explained by reduced exposure to light and other zeitgebers in older adults. However, studies have found that, when compared to young adults, older adults are exposed to at least as much bright light (e.g., in San Diego [5,6]) and to environmental and social zeitgebers of even greater regularity [7]. Nonetheless, it is likely that retinohypothalamic neurotransmission of light to the SCN is compromised in older adults due to glaucoma, macular degeneration, senile miosis, and other eye problems [8,9]. Moreover, age-related neurodegeneration of the suprachiasmatic nuclei (SCN) [10] could make the SCN less responsive to light in older adults. Preliminary evidence suggests that aging subjects may display smaller phase shifts to light stimuli [11,12]. Thus, older adults might require increased exposure to light or other synchronizers for adequate circadian entrainment.

Although light exposure is apparently the most important circadian synchronizer, careful regulation of the sleep-wake schedule [13,14], as well as physical activity [15,16] and social interaction [17], can also influence circadian timing. These non-photic stimuli can produce effects added to those produced by light alone.

Appropriately timed exposure to bright light and other zeitgebers might help correct circadian malsynchronization and alleviate sleep and mood problems that are common in older adults. Evidence indicates that light can also have photoperiodic effects on the organization of the human circadian system [18]. The main aims of this study were: (1) to contrast the influences of different bright light schedules on circadian and photoperiodic organization in older adults with insomnia and/or depression and in young, healthy controls; (2) to examine whether circadian "phase correction", i.e., shifting the circadian system to more normal timing, could improve sleep or mood among the older adults. However, since differing light treatments produced similar results, this presentation will emphasize the phase changes produced.

## Methods

### Subjects

Volunteers were 72 older adults (49 women, 23 men) ages 60–79 years, who were selected for complaints of insomnia and/or depression. The volunteers reported that their symptoms were of sufficient severity to warrant treatment. Volunteers were screened for freedom from melatonin-altering medications (with few exceptions), and the absence of acute health problems. A control group of 30 (n = 15 women, 15 men) young, healthy volunteers ages 20–40 years was studied in parallel. Volunteers signed their consent to participate in the study as approved by the UCSD Institutional Review Board.

### Home Baseline Procedures

Volunteers were first assessed for five-seven days in their home environments. Volunteers were asked to maintain their usual sleep and lifestyle habits during this time.

#### Baseline Urine Collection

Over two 24–30-hour periods (usually days three-four and six-seven at home), volunteers collected their urine samples approximately every two hours during wakefulness plus all voidings during the nighttime sleep period. Volunteers recorded the timing and volume of each collection, and stored 2-ml samples in their freezers. The samples were subsequently transferred to a laboratory -70°C freezer.

#### Baseline Sleep Assessment

An actigraph with minute-by-minute recordings of wrist activity and illumination was worn throughout home recording, except for short removals for bathing, etc. (Actillume I, Ambulatory Monitoring, Ardsley, New York). The nocturnal sleep periods were determined from actigraphic sleep and illumination recording combined with daily sleep diary data. Objective sleep was scored with a validated algorithm associating wrist movement with electroencephalographically-recorded sleep [19]. For each night, actigraphically-assessed sleep onset latency (SOL), total sleep time (TST), time spent awake after initial sleep onset (WASO), and sleep efficiency were determined. Each morning, subjective ratings of minutes of TST and WASO, and a 100 mm visual analogue rating of insomnia were also recorded. Mean baseline sleep levels were calculated, and have been reported previously [1].

#### Baseline Mood Assessment

The subjects' depressed moods were assessed on two days (usually days three and six) with the Center for Epidemiologic Studies-Depression (CES-D) questionnaire, which consists of 20 questions with four-point Likert responses (possible range: 0 to 60) [20]. The questionnaire was completed four hours after arising. Mean baseline CES-D was calculated. These data have been reported previously [1].

### Laboratory Procedures

**Figure 1:**
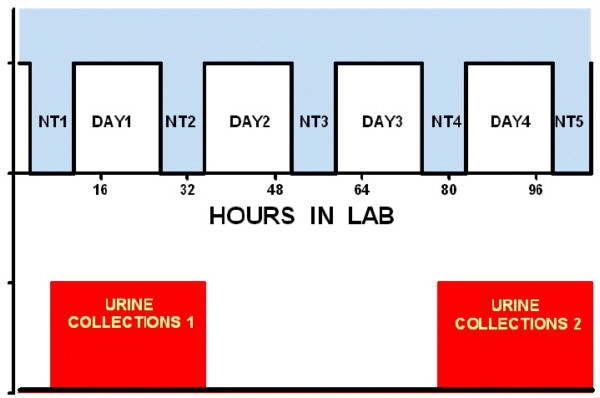
**Laboratory protocol**. Arriving two hours before their usual bedtime, subjects spent five nights and four days in the laboratory. This figure displays the time of urine collections (shown in red), which began after the last voiding before morning (most participants urinated during the night) and continued through the final morning voiding after the next consecutive night, slightly more than 24 hours.

Immediately following home recording, volunteers spent five nights and four days in the Circadian Pacemaker Laboratory at UCSD, arriving on Sunday evening two hours before bedtime, and leaving on Friday morning after arising (Figure 1). Each volunteer stayed in an individual apartment equipped with a bed, comfortable chair, television, kitchen, and private shower and bathroom facilities. Volunteers were provided with food of their own choosing (except for alcohol and caffeine) and were free to prepare and eat food ad libitum during the wake periods.

Sleep periods were fixed at eight hours, timed to correspond approximately with each volunteer's average home-recorded sleep schedule. Illumination levels were <0.5 lux during the sleep periods and ≤50 lux average in the horizontal direction at eye level during the 16-hour periods of wakefulness (except during the bright light treatments, described below). Sleep was discouraged during the wake periods with the aid of video monitoring, though some volunteers fell asleep for brief time periods (approximately two-five minutes). Volunteers were permitted one cup of coffee during the first four hours after arising. Vigorous exercise was not permitted, but light calisthenics and slow walking were allowed. Otherwise, volunteers were free to do what they wished during the wake periods, i.e., watch TV, receive visitors, read, etc. Because priority was placed on assuring that the laboratory experience did not trigger more severe depression, the laboratory staff made special efforts to help the volunteers feel comfortable and engaged in the laboratory experience. It was not uncommon for staff to spend several hours per day playing board games or chatting with a volunteer.

#### Light Treatments

**Figure 2:**
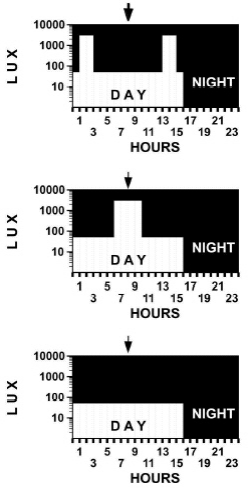
**Experimental Light Treatments**. Volunteers were randomly assigned to three four-hour light treatments (detailed in this figure) administered on four consecutive days against a background of <0.5 lux during eight-hour sleep periods and 50 lux during 16-hour wake periods. Treatment A was two hours at 3,000 lux from 1–3 hours and 13–15 hours after arising. Treatment B was four hours at 3,000 lux from 6–10 hours after arising. Treatment C was four hours of dim red light placebo from 6–10 hours after arising. Note that the center of each treatment was eight hours after arising, and the abscissa was hours after usual wake time.

Volunteers were randomly assigned to one of three light treatments, which were administered for four hours during each of the four days of the experiment (Figure 2). The light treatments were administered via overhead cool-white fluorescent lights, providing relatively even light levels at eye-level throughout the laboratory rooms.

**Treatment A **consisted of two hours of bright light (3,000 lux in the direction of gaze) at one-three hours after arising, as well as at three to one hours before bedtime. The 3000 lux portion of Treatment A was designed to resemble a skeleton LD14:10-long photoperiod. An LD16:8 skeleton might have had greater photoperiodic effect, but there was concern that LD16:8 might disturb sleep excessively. **Treatment B **consisted of four hours of bright light (3,000 lux) in the middle of the wake period, i.e., six-ten hours after awakening. The bright portion of Treatment B might resemble a short LD4:20 photoperiod, to the extent that the surrounding 50 lux treatment was photoperiodically ineffective. Also, bright light in Treatment B would be expected to fall in a relatively insensitive zone of the light phase response curve. **Treatment C**, the control treatment, involved four hours of placebo dim red light (1 lux) given six-ten hours after arising. With no bright light, it was expected that Treatment C would have little circadian effect. All treatments were superimposed upon the 16^th ^hour of background illumination of 50 lux. As observed in Figure 2, the center of timing of each light treatment was precisely eight hours after arising.

Volunteers were given standardized instructions designed to minimize potential differences in expectancy for beneficial effects of the treatments. After the volunteers were assigned to the treatments, expectancy for improvement in mood and sleep during the experiment was assessed via 100 mm visual analogue scales.

#### Urine Collection

As during home recording, urine was collected every two hours during wake and for any nighttime voidings. The collection time was over two periods of approximately 30 hours: from the last voiding during night one until wake-time on day two, and from the last voiding on night four until wake-time at the end of night five (see Figure 1).

#### Sleep Assessment

For each laboratory sleep period, measures of SOL, TST, WASO, and sleep efficiency were recorded and scored with standard polysomnographic procedures [21] as well as with actigraphy. In addition, subjective measures of TST, WASO, and insomnia were recorded each morning with diaries, as during home recording.

#### Mood Assessment

On day four, the subjects' depressed moods were assessed with the CES-D [20] four hours after arising. This represented the final CES-D score.

### Assays

Urinary concentrations of 6-sulphatoxymelatonin (aMT6s), the primary metabolite of melatonin, were assayed with a highly specific RIA assay developed by Aldous and Arendt (ALPCO, Ltd., Windham, NH, USA) [22]. Sensitivity of the RIA technique was <0.2 ng/mL. Intra- and inter-assay coefficients of variation were 3.3% and 6.7%, respectively.

### Data Analysis

#### Circadian Phase Assessment and Exclusion of Data

An investigator (JAE) used a four-point ranking system to rate the visual "quality" of the aMT6s excretion profiles: "excellent", "good", "poor", or "insufficient data". The ratings were based, for example, on whether the profiles had the expected patterns of transitions between daytime and nighttime levels, or whether higher or irregular baseline levels or abrupt spikes were observed. Artifacts might be attributable to many factors, including incomplete voiding of the bladder and inaccuracies in recording urinary volume or timing. Only data that were rated "excellent" or "good" were used for fitting 24-hour cosines to the aMT6s excretion data for estimation of aMT6s acrophase (24-hour fitted peak). Since estimation of circadian phase shift required assessment of both baseline and final circadian phases, the present analyses included only volunteers with aMT6s excretion profiles rated "excellent" or "good" for both baseline and final assessments. This reduced the number of older volunteers included in the analysis to 60, whereas aMT6s data could be analyzed in all 30 of the young volunteers.

**Figure 3:**
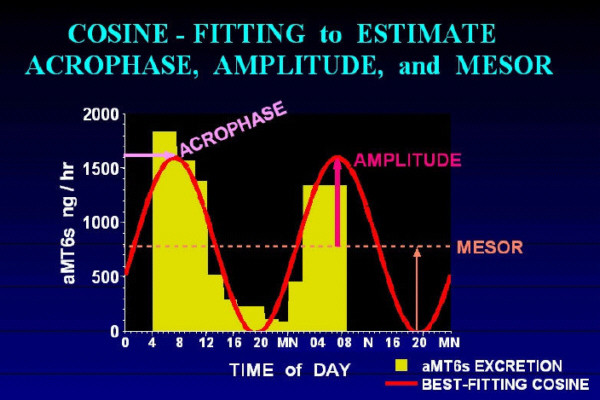
**Determining Urinary aMT6s Acrophase**. An example of analyzing urinary aMT6s is shown. The yellow area shows that the excretion rate of aMT6s from one voiding to the next was associated with each interval between voidings. The red line shows that a best-fitting cosine curve was estimated. The salmon dotted line indicates the mesor (the mean level of the fitted cosine). The rose arrow shows that the amplitude of the rhythm is the level of the peak of the fitted cosine above the mesor. The lavender arrow shows that the acrophase is the time of the peak of the fitted cosine referenced to the prior midnight.

**Baseline aMT6s acrophase **was estimated from data of the best quality. If both aMT6s profiles were of sufficient quality, a 24-hour cosine was fit across data for both days. If only one of the aMT6s profiles was of sufficient quality, the cosine was fit only for this day (e.g., Figure 3). If neither home profile was of sufficient quality, then baseline phase was defined by the aMT6s acrophase derived from day one in the laboratory (if this profile was of sufficient quality). In profiles of good quality, the home and first laboratory acrophases only differed, on average, by 0.03 hours. Baseline aMT6s acrophase was compared across treatment and age group via 3 × 2 ANOVA. **Final aMT6 acrophase **was determined from urinary data collected during the final 24–30 hours in the laboratory. The aMT6s parameters reflected the melatonin profile in the presence of light masking, both at home and in the laboratory.

#### Treatment Phase-Shifting Effects

According to convention, circadian phase shifts following the light treatments were calculated by subtracting the final aMT6s acrophase from the baseline aMT6s acrophase. Thus, negative and positive shifts indicated phase delays and phase advances, respectively. **Phase-response plots **were derived by plotting resultant circadian phase shifts (y-axis) against the circadian timing of the light treatments (A, B, or C) relative to the subjects' baseline aMT6s acrophases. The phase reference used for all light treatments was the center of the four-hour treatment, which was also the center of the 16-hour period of background illumination and wakefulness. Within the restricted phase range, the phase response data was sufficiently linear for slopes and elevations of linear regression lines to be compared via ANOVA, using procedures described by Zar [23].

#### Circadian Abnormality and Phase Correction

Two measures of circadian misalignment were calculated. First, since our previous analysis of this group indicated that sleep was best when the aMT6s acrophase coincided approximately with mid-sleep (i.e., mid-point between lights out and final time of awakening) [1], **circadian malsynchronization **was defined as the absolute phase angle (h) between an individual's aMT6s acrophase and his/her mid-sleep. Second, **circadian phase dispersion **was defined as the absolute number of hours between an individual's aMT6s acrophase and the median aMT6s acrophase for his/her age group. These measures were calculated for both baseline and final aMT6s acrophase data. Baseline measures of circadian malsynchronization and phase dispersion were compared via 3 × 2 treatment-by-age group ANOVAs. Two measures of **circadian phase correction **were defined by the extent to which circadian malsynchronization and phase dispersion were decreased from baseline to final phase assessment. These changes were compared with 3 × 2 × 2 light treatment-by-age group (older vs. young)-by-time (baseline vs. final) ANOVAs.

#### Treatment Effects on Mood and Sleep

Mean home baseline CES-D data was compared with CES-D responses during the final day in the laboratory. The laboratory actigraphic data was regarded as the most relevant sleep data to compare to baseline, because actigraphic data allowed comparisons of objective home versus laboratory sleep. To assess changes in sleep associated with the treatments, mean home actigraphic data was compared with mean actigraphic data from the final two nights in the laboratory. Likewise, mean home sleep diary data was compared with the mean diary reports of the last two nights in the laboratory. Analysis of polysomonographic data compared data averaged across the first two nights in the laboratory to the last two nights in the laboratory. Changes in mood and sleep following the treatments were assessed via 3 × 2 × 2 treatment-by-age group-by-time ANOVAs.

#### Association of Phase Correction with Changes in Mood and Sleep

The association of changes in circadian malsynchronization and phase dispersion with changes in sleep and mood following treatment were assessed in two ways. First, Spearman rank-order correlations were calculated. Second, t-tests compared changes in sleep and mood between groups that had phase correction versus groups that had no phase correction (i.e., had no change or increases in malsynchronization and phase dispersion).

## Results

### Circadian Timing

As measured by Actillume in the week before entering the laboratory, the center of the sleep periods averaged 03:20 at home. In the laboratory, measurments mid-dark averaged 03:11 (a small but significant difference: p < 0.025). As measured by Actillume, the median mesor illumination (24-hour fitted mean) was 478 lux at home and 349 lux, 381 lux, and 30 lux respectively for treatments A, B, and C in the laboratory. However, the acrophases of 24-hour Actillume illumination measured in lux were 13:09 at home and 15:58 in the laboratory, reflecting the tendency of bright daylight exposures at home to occur before mid-wake.

### Baseline and Final aMT6s Acrophase Data

The aMT6s data for the older and young volunteers are displayed in Table 1. No significant treatment, age group, or treatment-by-age group interaction effects were found for aMT6s acrophase data. Likewise, no significant treatment effects were observed for the final laboratory aMT6s mesor or the estimated duration of aMT6s secretion (data not shown).

**Table 1:** aMT6s acrophase and measures of circadian malsynchronization and phase dispersion in older (n = 60, ages 60–79) and young volunteers (n = 30, ages 20–40), mean and SE.

Age Group	Baseline aMT6s Acrophase	Final aMT6s Acrophase	Baseline Circadian Malsynch.	Final Circadian Malsynch.	Baseline Circadian Dispersion	Final Circadian Dispersion
Older	4.01 ± 0.25	4.68 ± 0.28	1.57 ± 0.16	2.19 ± 0.19	1.53 ± 0.15	1.60 ± 0.18
Young	4.14 ± 0.23	5.07 ± 0.28	0.69 ± 0.11	1.09 ± 0.23	1.04 ± 0.12	1.10 ± 0.20

### Treatment Phase-Shifting Effects

**Figure 4:**
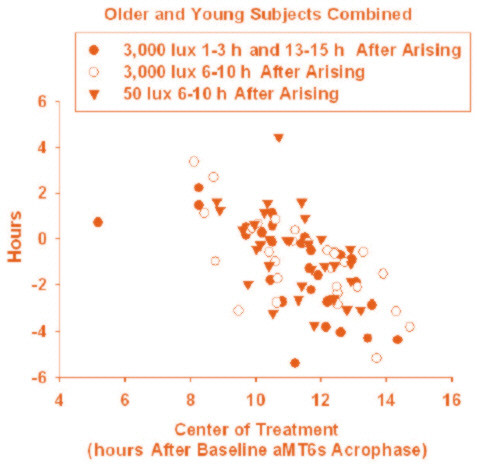
**Phase Response Plots for each Light Treatment**. Shown are the shifts in aMT6s acrophase, which varied significantly for each treatment, as a function of the circadian timing of the light treatments, defined as the center of treatment (eight hours after arising) relative to the aMT6s acrophase at baseline.

Phase responses to the treatments are displayed in Figure 4. Significant linear regressions associating the circadian timing of the light treatments with shifts in aMT6s acrophase were found for each treatment. However, there was no significant difference between treatments in the slopes or in the origins of the regression lines. Across all treatments, there was a significant mean delay in aMT6s acrophase from baseline to final assessment (45 min ± 15 min SEM, t = 3.04, p = 0.003); however, there were no significant treatment-by-time or age group-by-time interaction effects.

### Circadian Abnormality and Phase Correction

**Figure 5:**
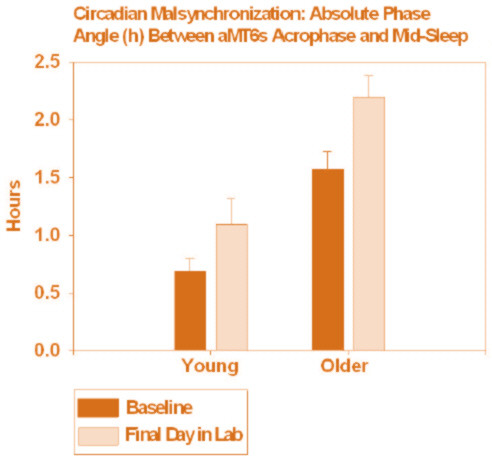
**Circadian malsynchronization at baseline and final assessment**. Shown is circadian malsynchronization, defined as the absolute phase angle (mean ± SE hours) between aMT6 acrophase and mid sleep, determined at baseline and following the light treatments. A significant increase in malsynchronization was found.

**Figure 6:**
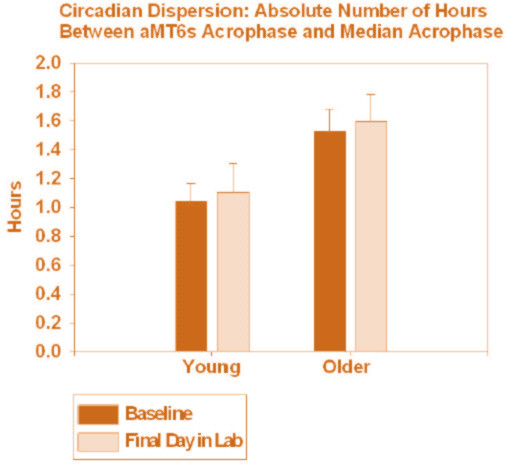
**Phase dispersion at baseline and final assessment**. Shown is phase dispersion, defined as the absolute number of hours (mean ± SE) between aMT6s acrophase and the median aMT6s acrophase, determined at baseline and following the treatments.

As compared to younger subjects, at baseline the older subjects had more circadian malsynchronization [t(1,88) = 4.57, p < 0.001] and greater circadian phase dispersion [t(1,88) = 2.50, p = 0.014]. However, there were no significant treatment or treatment-by-age group differences between these variables at baseline (before treatment). There was a significant increase in circadian malsynchronization from baseline to final assessment [F(1,88) = 8.5, p = 0.004] (Figure 5), indicating the delays in aMT6s acrophase. However, there was no significant treatment-by-time or age group-by-time interaction in this effect. Circadian phase dispersion showed no significant change over time (Figure 6), and no significant treatment-by-time or age group-by-time interaction.

### Treatment Effects on Mood and Sleep

Volunteers reported equal expectancy for improvements in sleep and mood following each treatment. A significant reduction in the CES-D from baseline to final measurement was found [F(1,82) = 13.8, p < 0.001]. There was no treatment-by-time interaction for CES-D. A near-significant age-group-by-time effect was found for CES-D (F(1,82) = 3.7, p = 0.058]. CES-D was reduced from 15.4 ± 1.1 to 11.4 ± 1.0 in the older group and from 9.1 ± 1.5 to 8.0 ± 1.3 in the younger group.

In actigraphic data, significantly less TST, lower sleep efficiency, and greater WASO were found in the older volunteers as compared to the young volunteers. A significant age group-by-time effect for actigraphic TST was mediated by slight increases from baseline to final assessment in the older group (from 334.3 ± 8.8 min to 339.3 ± 7.6 min) but decreases in the young group (from 450.8 ± 9.4 min to 367.6 ± 7.7 min). No significant treatment or treatment-by-time effects for actigraphic sleep were found.

In sleep diary measures, significantly less TST and significantly greater WASO and insomnia (100 mm visual analogue) were found for the older volunteers in comparison to the young volunteers. A significant age group-by-time interaction for insomnia was mediated by decreases in the older group (from 44.7 ± 1.9 mm to 39.5 ± 2.8 mm) and increases in the young group (from 15.3 ± 2.6 mm to 19.8 ± 3.5 mm.) No significant treatment or treatment-by-time effects for these variables was found.

The older group had significantly less polysomnographic TST and more WASO compared with the young group. However, no significant age group contrasts by time, treatment, or treatment-by-time interaction were found for polysomnographic sleep.

### Correlations of "Phase Correction" with Changes in Mood and Sleep

Changes in circadian malsynchronization and phase dispersion were not significantly correlated with changes in mood or sleep. Moreover, changes in mood and sleep were not different between individuals who experienced decreases in circadian malsynchronization or decreases in phase dispersion following treatment compared with those who experienced no phase correction.

## Discussion

Surprisingly, no significant contrasts between the three light treatments were demonstrated for melatonin or actigraphic parameters, sleep, or mood, nor could changes in circadian misalignment be associated with disturbances of sleep or mood. Perhaps a longer period of randomized treatment would have resulted in greater contrasts, but significant effects of bright light treatment on mood have been demonstrated with treatment of one week or less, and it is not currently clear that treatment beyond one week produces any greater benefit [24]. Probably, 10,000 lux would have been slightly more effective than 3,000 lux. Urine collections of aMT6s are not the most precise method of estimating melatonin timing, but they were considered less burdensome on the depressed participants than alternative methods.

Significant phase-responses were evident after each laboratory treatment. The average delay in aMT6s acrophase in the laboratory was probably due to the dimmer and later illumination timing in the laboratory as compared to the home situation, because illumination peaked before mid-wake at home, mid-wake averaging well after noon. The similarity in phase responses between the two bright light treatments was consistent with previous studies, suggesting that phase-shifting effects may be estimated from the timing center of light treatments [25,26]. However, it might have been predicted that treatment A would fall on a more active portion of the phase-response curve than treatment B. Also, the remarkable similarity in the phase responses for the dim placebo treatment in comparison to the bright light treatments was unexpected. Dose-response studies have indicated that phase-shifting effects of light are related to cube-root [27] or logistic functions [28] of illumination, either of which would predict that the bright light treatments (3,000 lux) were at least two-fold stronger than the placebo treatment (50 lux). Nonetheless, it appears that the dim placebo and bright light treatments had similar phase-shifting potency in the present protocol.

Our phase shift responses could be explained by nonphotic zeitgebers, including the imposed sleep-wake cycle, social cues, and activity/rest. There is evidence that the sleep-wake cycle is a potent zeitgeber; this effect may be independent of the light-dark cycle [13,29,30]. Combining a fixed sleep-wake routine, with appropriately timed bright light, produces additive phase-shifting effects [13,29]. Both classic and recent research has shown that social interaction can be a significant zeitgeber [17,29,31,32].

Particular procedures used in the present study might have facilitated non-photic entrainment. For example, during the baseline week, volunteers in the present study were asked to maintain their usual sleep-wake and daily routines. These routines were often quite erratic, which might have rendered the circadian system more sensitive to the fixed high amplitude rest/activity schedule in the laboratory. In other studies, subjects have been required to maintain more rigid baseline sleep-wake schedules prior to experimental treatment [27,28].

The degree of social interaction between the volunteers and staff in the present study was greater than that which was permitted in many other studies. Laboratory social interaction was designed both to provide comfort and to help monitor the volunteers for safety. Social interaction may have significant independent zeitgeber effects, and can act synergistically with light exposure. Clinically, it is relevant to examine light effects in the presence of social interaction.

The equivalent reduction in depression following each treatment did not support the prediction of greater antidepressant effects with bright light. Possibly the influence of light could be attenuated by the kind care the volunteers received in the laboratory, the social interactions, and placebo effects. Moreover, the light treatment was for fewer days than that employed in the majority of clinical trials of bright light treatment [24], so an insufficient duration might explain the lack of significant effect. An insufficient duration or intensity of light treatment might also explain the failure to observe photoperiodic effects on the duration of aMT6s excretion.

Another unexpected finding was the significant increase in circadian malsynchronization following the bright light treatments. Phase dispersion also showed a non-significant increase. The phase-response plots indicated that the treatments resulted in "over-corrections" of circadian phase. Volunteers with the most advanced body clocks in reference to sleep at baseline (whose light treatment was therefore centered more than 12 hours after the aMT6s acrophase) demonstrated large phase delays as shown in Figure 4. Conversely, those most delayed in reference to sleep at baseline experienced large phase advances. The corrections were often greater than the amounts of initial phase abnormality, contrary to hypothesis. Also, reductions in circadian malsynchronization or phase dispersion (phase correction) were not correlated with improvements in sleep and mood. Chronic mood and sleep problems associated with circadian malsynchronization might be difficult to correct in such a short period of time, although we had expected to find measurable responses.

## Conclusion

Consistent with previous studies, compared to young adults, older adults had significantly greater circadian malsynchronization and phase dispersion. Significant and remarkably similar phase-responses were found for each of the three light treatment schedules. The results suggest that low levels of illumination and/or fixed timing of behavior had significant circadian phase-shifting effects. The large phase-shifts resulted in a significant increase in circadian malsynchronization, rather than phase correction. Moreover, phase correction was not significantly associated with improvements in sleep or mood.

## Competing interests

The author(s) declare that they have no competing interests.

## Authors' contributions

SDY supervised the data collection, subject recruitment, data analysis, and drafting the manuscript.

DFK conceived of the study and was a principal investigator, screened the subjects, and assisted in data analysis and drafting of the manuscript.

JAE assisted in designing the study and drafting the manuscript and performed the aMT6s assays.

KMR assisted in designing the study, in laboratory data collection, and in drafting the manuscript.
